# Contactless Inductive Sensors Using Glass-Coated Microwires

**DOI:** 10.3390/s26020428

**Published:** 2026-01-09

**Authors:** Larissa V. Panina, Adrian Acuna, Nikolay A. Yudanov, Alena Pashnina, Valeriya Kolesnikova, Valeria Rodionova

**Affiliations:** 1 Department of Materials Technology of Electronics, National University of Science and Technology (MISiS), 119049 Moscow, Russia; aacuna@uclv.cu (A.A.); yudanov.n@misis.ru (N.A.Y.); m2007687@edu.misis.ru (A.P.); 2 REC Smart Materials and Biomedical Applications, Immanuel Kant Baltic Federal University, 236004 Kaliningrad, Russia; vgkolesnikova1@kantiana.ru (V.K.); vvrodionova@kantiana.ru (V.R.); 3 Department of Electronics and Telecommunications, Faculty of Electrical Engineering, Central University “Marta Abreu” of the Villas (UCLV), Santa Clara 50100, Cuba

**Keywords:** amorphous microwires, contactless sensors, nonlinear response, harmonic analysis, magnetic bistability, stress/strain detection, temperature sensing, inductive spectroscopy

## Abstract

**Highlights:**

**What are the main findings?**

- Tunability of magnetic properties of amorphous microwires by composition and annealing helps to realize a specific nonlinear magnetization response.
- The extent and nature of magnetic nonlinearity is controlled through external stimuli.

**What are the implications of the main findings?**

- Amorphous and nanocrystalline microwires are used for contactless sensors based on nonlinear magnetization dynamics.
- Two key contactless readout methodologies: time-domain detection of the switching field for bistable wires and frequency-domain harmonic analysis are used for sensor output.
- Contactless mechanical stress, pressure, temperature sensors and magnetic particle detection are discussed.

**Abstract:**

This paper explores the potential of amorphous and nanocrystalline glass-coated microwires as highly versatile, miniaturized sensing elements, leveraging their intrinsic nonlinear magnetization dynamics. In magnetic systems, this approach is particularly advantageous because the degree of nonlinearity can be externally tuned using stimuli such as applied magnetic fields, mechanical stress, or temperature variations. From this context, we summarize key properties of microwires—including bistability, a specific easy magnetization direction, internal stress distributions, and magnetostriction—that can be tailored through composition and annealing. In this review, we compare for the first time two key contactless readout methodologies: (i) time-domain detection of the switching field and (ii) frequency-domain harmonic analysis of the induced voltage. These principles have been successfully applied to a broad range of practical sensors, including devices for monitoring mechanical stress in structural materials, measuring temperature in biomedical settings, and detecting magnetic particles. Together, these advances highlight the potential of microwires for embedded, wireless sensing in both engineering and medical applications.

## 1. Introduction

The development of contactless sensors is increasingly important across many technological fields, ranging from non-destructive testing to personalized medicine. A standard approach to energy harvesting and data communication relies on microwave transducers with antennas, piezoelectric transducers, magnetoelectric transducers, and resonant inductive circuits [1,2,3,4,5]. However, the widespread deployment of these systems is often constrained by the power consumption and physical size of the sensing elements. From this perspective, short-range interrogation using inductive sensing technology can be particularly advantageous. In this paper, we focus on inductive wireless sensing that exploits the nonlinear magnetization of soft magnetic materials.

Magnetic induction technology forms the foundation of various sensing platforms, including search coils, coupled resonance coils, fluxgate sensors, and magnetoimpedance (MI) sensors [6,7,8,9,10,11]. These systems leverage both linear and nonlinear magnetization dynamics. In particular, nonlinear dynamics presents distinct advantages [12], especially when employed in conjunction with spectral analysis techniques for signal detection and characterization. This approach enables improved performance in terms of sensitivity, resolution, and robustness against environmental noise.

In nonlinear magnetic systems, a periodic excitation field—typically sinusoidal or triangular—is applied to the sensing element to drive its magnetization between positive and negative saturation. The resulting system response is analyzed in terms of deviations from the excitation waveform, revealing information about the magnetic material’s behavior under dynamic conditions. Spectral analysis serves as a versatile and powerful tool for investigating such nonlinear responses. In magnetic systems, this approach is particularly advantageous due to the ability to externally control the degree of nonlinearity through stimuli such as applied magnetic fields, mechanical stress, or temperature variations.

In fluxgate sensor configurations, the presence of a target magnetic signal alters the system’s power spectral density, most notably through the emergence of even harmonics of the excitation frequency (ω). The second harmonic (2ω) is commonly used for signal detection and quantification due to its sensitivity to external magnetic fields. Although the introduction of an asymmetrizing DC bias can amplify this effect, such an approach may be unsuitable for certain applications, particularly where low power consumption or a symmetric response is required. As an alternative, readout schemes based on higher-order harmonic analysis—typically implemented via lock-in amplification—can provide enhanced sensitivity without the need for biasing. Furthermore, various frequency-mixing techniques have been investigated as a means of improving signal extraction and overall sensor performance [13,14,15].

For sensors employing magnetically bistable materials, an alternative event-based approach can be utilized. In this method, the sensor output is analyzed in the time domain by capturing discrete magnetization switching events triggered by external stimuli [16,17,18]. This time-resolved detection mechanism has facilitated the development of wireless sensing systems with glass-coated magnetic microwires [19] serving as a particularly effective material platform due to their strong bistability, high sensitivity, and compact form factor.

Amorphous materials produced by rapid melt quenching in the form of wires and ribbons exhibit not only excellent soft-magnetic behavior, but also favorable mechanical properties and small dimensions. Glass-coated microwires typically possess a metallic core diameter of 1–50 µm and a glass coating thickness of 3–15 µm [20,21,22]. Owing to the absence of magnetocrystalline anisotropy, magnetostriction becomes the principal parameter for tuning the magnetic properties. By selecting appropriate compositions of ferromagnetic elements such as Fe, Co, and Ni it is possible to obtain wires with magnetostriction $\lambda_{s}$ spanning from relatively large positive values to nearly zero and negative values [23]. Increasing the metalloid (Si, B) content above ~25 at.% significantly reduces both the Curie temperature and the saturation magnetization [24].

Amorphous ferromagnetic microwires with positive magnetostriction exhibit pronounced nonlinear magnetization behavior, characterized by magnetic bistability [25,26,27]. In such materials, only two stable magnetic states—corresponding to magnetization values of $\pm M_{s}$—are realized. Transitions between these states occur abruptly at a specific external magnetic field strength, known as the switching field ($H_{SW}$). This sharp switching behavior arises from the wire’s internal domain structure, which consists of a central core domain with axial magnetization and outer shell domains with radial or circular magnetization. The value of $H_{SW}$ is highly sensitive to external parameters such as excitation conditions, temperature, and mechanical stress, making it a key parameter for sensing applications. When a triangular excitation magnetic field is applied, the switching field can be directly mapped to a corresponding switching time, allowing for straightforward time-domain signal processing. The use of glass-coated microwires exhibiting magnetic bistability has proven particularly effective in the development of compact, wireless sensors for monitoring mechanical stress [17,18,28,29], temperature [30,31,32], and position [33,34].

In materials with a transverse easy-axis anisotropy relative to an applied axial magnetic field the magnetic susceptibility is high for driving fields lower than the saturation field. Such magnetic structure is typical of negative magnetostriction microwires exhibiting a circular easy magnetization. As the magnetization approaches saturation, the susceptibility drops sharply to zero, producing distinct harmonic signatures [35,36,37]. The characteristic field required to reach saturation is governed by the effective anisotropy field, which, in amorphous alloys, is strongly influenced by mechanical stress due to the dominant role of magnetoelastic anisotropy. Then, analyzing harmonic spectrum it is possible to measure the effective anisotropy field, and consequently, mechanical stress. Wireless sensors based on spectral analysis have advantages over resonant inductive sensors with magnetostrictive components, as the output signal can be made independent on detection distance by using several harmonics amplitudes.

This paper revisits the use of intrinsic nonlinear magnetization dynamics in amorphous microwires as a foundation for developing novel measurement systems and readout methodologies. We also present new results for temperature monitoring and magnetic particle detection using amorphous microwires with negative magnetostriction. The structure of the paper is as follows: Section 2 discusses spectral analysis techniques and specific detection methods that exploit nonlinear magnetization behavior. Section 3 reviews the key properties of glass-coated microwires relevant to sensing applications. Section 4 presents selected examples demonstrating the practical implementation of these materials in various sensor applications.

## 2. Spectral Analysis Techniques

Transfer function of a periodically excited system,

(1) $$V\left(x\left(t\right)\right)=V\left(x\left(t+T\right)\right),$$

is always only composed of discrete harmonic contributions with $\omega =n\omega_{o}$, ωo=2πT, no matter if the excitation $x\left(t\right)\;$ itself is harmonic or not [13,38]. In the course of a Fourier analysis, the periodic signal $V\left(t\right)$ is decomposed into its harmonic constituents:

(2) $$V\left(t\right)=\sum A_{n}\cos\left(2\pi f_{0}nt\right)+B_{n}\sin\left(2\pi f_{0}nt\right),$$

yielding the amplitudes $A_{n}$ and $B_{n}$. In this work, we focus on the induced voltage generated by magnetization change, so the signal is proportional to $dM\left(t\right)/dt$. The time derivative introduces a factor of $\omega_{0}n$ to each Fourier component. In other words, it amplifies the higher harmonic contributions in proportion to n. Essentially, the spectral analysis represents a universal approach for investigating nonlinear systems. In magnetic systems, however, the specificity arises from the ability to externally control nonlinear behavior through factors such as applied magnetic fields or mechanical stress.

**Figure 1:**
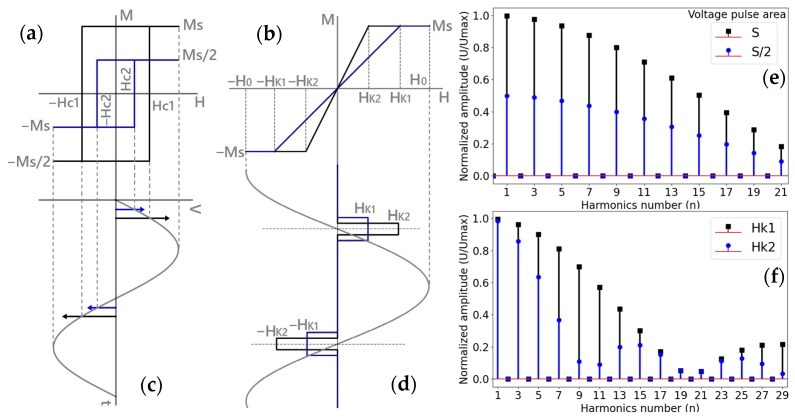
Magnetization scenarios of soft magnetic materials. (**a**,**b**)—types of magnetization loops, (**c**,**d**)—characteristic induced voltage pulses, (**e**,**f**)—examples of spectral amplitudes for magnetization types of (**a**) and (**c**), respectively. In (**c**), the arrows represent the sharp generated voltages. For (**e**), the spectral data were calculated by assuming that the area *S* under the rectangular voltage pulse decreased twice due to the corresponding change in the saturation magnetization (for example, when temperature is increased and approaching to the temperature of the paramagnetic state transition known as the Curie temperature, $T_{c}$). In (**f**), the pulse change is due to increase in the anisotropy field $H_{K}$ ($H_{K1}$ > $H_{K2}$).

Soft magnetic materials are distinguished by low values of the anisotropy field ($H_{K}$) and coercivity field ($H_{c}$), typically ranging from 100 to 200 A/m, and even lower. The composition and geometry of soft magnetic materials can be tailored to control their magnetic structure, thereby resulting in a broad spectrum of magnetization behaviors and observed hysteresis loops. In essence, considering ferromagnetic wires subjected to a magnetic field applied along their axis, the resulting hysteresis loops can be categorized into two distinct types. The first type is characterized by a close-to-rectangular loop, which is attributed to an axial easy magnetization direction. The second type is nearly non-hysteretic, resulting from specific easy magnetization along circumference. The corresponding circumferential anisotropy field in turn determines the saturation regime. These are shown in Figure 1a,b, respectively.

In the first scenario, magnetization proceeds via rapid and abrupt domain wall propagation at a critical field, commonly referred to as the switching field $H_{SW}$. In the second scenario, magnetization occurs through rotation under a hard-axis magnetic field, without domain wall motion. When the characteristic fields such as $H_{c}$, $H_{SW},\;H_{K}$ are smaller than the amplitude $H_{0}$ of the magnetizing field H(t), the magnetization exhibits strong nonlinearity with respect to H(t), in both cases, leading to the appearance of higher-order harmonics in the induced voltage spectrum (see Figure 1c–f). The shape of the hysteresis loop and the critical fields are influenced by physical parameters, such as mechanical stress, strain, temperature, etc. [22,39] which can be quantitatively assessed by analyzing the voltage spectrum.

Thanks to the inductive sensing principle, remote interrogation is possible, enabling the use of sensing elements embedded within materials. For materials exhibiting rectangular hysteresis loops, the coercive field $H_{C}$ does not directly affect the amplitudes $A_{n}$ and $B_{n}$ of the harmonic components. Consequently, predicting changes in higher harmonics caused by variations in $H_{c}$ can be challenging. However, near the Curie temperature, remagnetization dynamics change significantly, and the harmonic amplitudes reflect temperature-dependent variations, as the area under the voltage pulse scales with the saturation magnetization $M_{s}$. A corresponding spectral example considering a twofold decrease in $M_{s}$ is illustrated in Figure 1e.

For non-hysteretic magnetization behavior, the characteristic anisotropy field $H_{K}$ governs the magnetic susceptibility χ, and the harmonic spectrum strongly depends on the ratio $H_{K}/H_{0}$. In the case of magnetization behavior approaching the idealized model shown in Figure 1b, the harmonic amplitudes $A_{n}$ and $B_{n}$ vary non-monotonically with increasing harmonic order n, exhibiting minima whose positions depend on $H_{K}/H_{0}$, as demonstrated in Figure 1f.

An intriguing scenario arises when external stimuli induce a change in the hysteresis type by altering the easy-axis anisotropy, leading to transitions such as from a rectangular to an inclined hysteresis loop. Such transitions typically result in a pronounced decrease in harmonic amplitudes due to modifications in the easy anisotropy [36,37].

Depending on the specific application, a DC bias magnetic field may be applied. First of all, this breaks the symmetry, leading to the emergence of even-order harmonics. It also causes a rapid decrease in the amplitudes of higher-order harmonics, and this has been proposed for the detection of magnetic particles and spatial mapping. This can be illustrated considering superparamagnetic particles detection [40,41]. This can be demonstrated considering their magnetization as a function of the field described by the Langevin function $L\left(x\right)$:

(3) $$M\left(T,H\right)=M_{s}L\left(x\right),L\left(x\right)=\coth\left(x\right)-\frac{1}{x},x=\frac{MH}{k_{b}T}.$$

The expansion of this function around the zero-field point $x_{0}$ = 0, gives

(4) $$L\left(x\right)\approx \left(x_{0}=0\right)\approx \frac{x}{3}-\frac{x^{3}}{45}+\frac{2x^{5}}{945}+{O\left[x\right]}^{7}.$$

In Equation (4) the nonlinear terms are considerably smaller than the linear one. However, in fields closer to saturation, the contribution of nonlinear behavior becomes more significant. Thus, for $x_{0}$ = 3 the expansion takes the form

(5) $$L\left(x\right)\approx -0.08+0.49x-0.11x^{2}+0.014x^{3}+{O\left[x\right]}^{4}.$$

As seen from Equation (5), nonlinearity in the form of the quadratic term is more than three times larger compared to the case when $x_{0}$ = 0. However, as the harmonic order *n* increases, the amplitudes of higher-order harmonics decrease much faster than in the absence of a bias field. By applying a bias magnetic field to modulate nonlinearity, it becomes possible to detect the spatial distribution of magnetic particles, a principle exploited in magnetic particle imaging (MPI). Specifically, with an appropriately chosen bias field, higher harmonics can effectively be “switched off” once the offset field strength exceeds a threshold, rendering these harmonic contributions undetectable against experimental noise. MPI’s first commercial products were available as early as 2013 [42], but more research and development is needed if MPI is to fulfill its potential and be used appropriately in clinical imaging [43].

### 2.1. Spectrum Measurement

**Figure 2:**
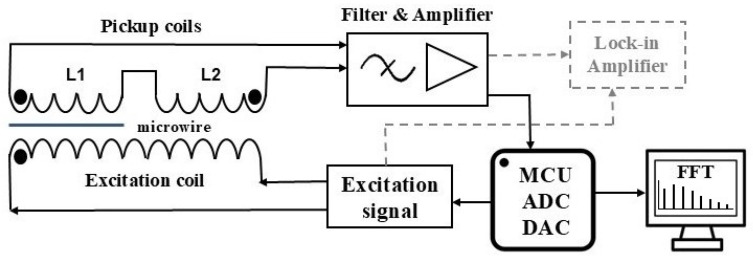
Schematics for measuring the harmonic spectrum of the voltage pulse induced in the magnetic element during remagnetization.

The voltage pulses generated by the microwire due to remagnetization can be measured using an inductive method, that employs two sets of coils: an excitation coil and a pair of differential pickup coils, as shown in Figure 2. The signal from the pickup coils is then subjected to a series of conditioning steps, including filtering and amplification. A lock-in amplifier is subsequently used to obtain the harmonic spectrum. The harmonic amplitudes are measured under the condition that the phase shift relative to the reference (excitation) signal is zero.

Additionally, the voltage pulse can be digitized using an analog-to-digital converter (ADC) integrated into a microcontroller unit (MCU). The harmonic amplitudes are then determined by applying a Fast Fourier Transform (FFT) to the discrete signal recorded by the MCU. The MCU also generates the excitation signal through its digital-to-analog converter (DAC). This approach enables the development of a compact sensor suitable for practical applications.

### 2.2. Frequency Mixing Method of Detection

The frequency mixing method has been used in various sensor applications for decades (see, for example, [13,14] for a general discussion of frequency mixing). This approach has recently been refined for the detection of superparamagnetic particles whose magnetization follows the Langevin Equation (3). It utilizes the simultaneous application of a low-frequency ($f_{1}$), high-amplitude magnetic field and a high-frequency ($f_{2}$), lower-amplitude field. This configuration enhances the nearest significant nonlinear term, as indicated by Equation (5). The induced voltage is then measured at combined frequencies of the form $mf_{2}\pm nf_{1}$, and through demodulation, a specific harmonic is isolated—typically with m=1 and n=2. Compared with other leading magnetic sensor–based detection techniques, this method enables the development of more cost-effective detection systems. In addition, because only nonlinear magnetic responses contribute to the output signal, the influence of surrounding materials is inherently canceled. Consequently, background interference is greatly minimized, resulting in high sensitivity and specificity for magnetic particle detection [44,45]. The response signal of the detection coil can be measured using a standard lock-in amplifier, as discussed in Section 2.1. Different concentrations of iron oxide nanoparticle suspensions were used to validate the detection technique and determine the limit of detection.

This technique was later proposed for measuring various internal parameters of magnetic materials that exhibit nonlinear magnetization behavior. In particular, it enables measurement of the magnetic susceptibility of materials with hysteresis loops similar to that shown in Figure 1b [46]. A low frequency magnetic field with an amplitude exceeding $H_{K}$ induces periodic saturation of the sample and periodic changes in its susceptibility χ from high to low values at twice the excitation frequency (2$f_{1}$). This field corresponds to the low-frequency field discussed above. The material under investigation is incorporated into an LC resonant circuit oscillating at a resonance frequency ($f_{res}\sim MHz$), which matches the frequency of an external oscillator when χ is small. As χ increases, the circuit inductance increases, resulting in a decrease in the resonance frequency. This leads to a phase difference between the external oscillator and the LC circuit, which is used as the output signal to measure changes in susceptibility. By applying a low-pass filter, only harmonics of 2$f_{1}$ are retained and analyzed using FFT. In materials with a smaller anisotropy field and high initial susceptibility, the harmonic amplitudes decrease slowly with increasing harmonic order. In contrast, materials with a higher anisotropy field exhibit a rapid decrease in harmonic amplitudes. The principle of frequency mixing was applied to non-contact strain measurements using a nanocrystalline ribbon (Vitroperm, containing Fe, Cu, Nb, B and Si [47]) with a magnetostriction constant of 0.2 ppm. It was also demonstrated that a combination of four harmonics of ${2f}_{1}$ could be used to perform distance-independent strain measurements at distances of 0.75 mm and 1.75 mm.

The frequency mixing approach was employed to detect the stress-sensitive microwave response of a single microwire exhibiting the giant magnetoimpedance effect [48,49]. For negative magnetostrictive wires, the impedance as a function of the DC magnetic field demonstrates a double-peak curve. When a microwire is subjected to GHz-frequency excitation and a low-frequency modulating field with $f_{mod}$ is applied, the measured *S*-parameters will be modulated at a frequency of ${2f}_{mod}$. This is related to the impedance dependence on permeability and its symmetry with respect to the modulating field. When the amplitude of the modulating field exceeds the anisotropy field, the $n{2f}_{mod}$ harmonic components can be detected as a function of the applied stress. Free-space microwave spectroscopy was used to measure the stress dependence of the $S_{22}$ parameter of a single cobalt-based glass-coated microwire at a frequency of 2.45 GHz using a vector network analyzer and broadband horn antennas. The modulating frequency was 80 Hz. The change in the $S_{22}$ parameter correlated with the stress dependence of the hysteresis loops and permeability.

### 2.3. Measuring the Switching Field in a Time Domain

**Figure 3:**
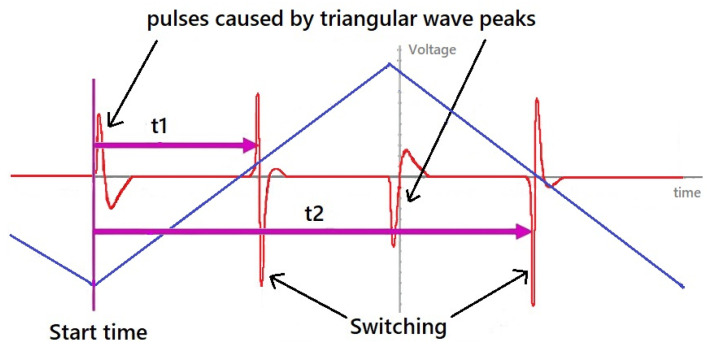
Schematics of switching field detection in a time domain. Triangular driving field waveform (blue lines) and induced voltages in the detection coil are shown. Four pulses are induced during one period. Two of them occur when the field suddenly shifts from increasing to decreasing after the apex of a triangle waveform. The other two correspond to the microwire switching. While the pulse arising from the slope change is used to start the timer, the second maximum from the wire is used to position detection.

For magnetic samples that exhibit bistability and are used as sensing elements, an alternative detection methodology based on measurements of the switching field $H_{sw}$ has been proposed. During remagnetization, very narrow voltage pulses are generated, and the magnetic field at which they are centered is defined as the switching field (Figure 1a,c). The value of $H_{sw}$ can be measured directly by using a magnetic field with a triangular waveform as the excitation source. In this case, $H_{sw}$ is expressed in terms of the switching time. Figure 3 illustrates this detection method: the instants at which the switching field is reached are referred to as the switching times ($t_{1}$ and $t_{2}$ in Figure 3). The average switching time represents the coercivity field. When an additional external magnetic field is present due to environmental noise, using $t_{1}+t_{2}$ eliminates its contribution. To accurately process the switching time, a suitable control unit capable of accurately recording this time is necessary [19].

A unique characteristic of bistable microwires is the periodic generation of the same signal in response to the applied magnetic field, mechanical stress, or surrounding temperature. Due to this property, it is possible to average signals from multiple periods. This eliminates unwanted background noise in the signal and allows for a more accurate measurement of the switching time. This method was successfully employed using magnetically bi-stable microwires of proper composition to measure stress/strain, temperature and position [28,29,30,31,32,33,34].

## 3. Magnetization Reversal in Ferromagnetic Amorphous Microwires: Effect of Stress and Temperature

Amorphous glass-coated microwires fabricated by the Taylor–Ulitovski method [20,21] constitute highly effective soft-magnetic materials for miniature sensing systems that exploit nonlinear magnetic behavior. The metal core diameter d and total diameter D can widely vary but in majority of applications d = 10–30 microns and D = 15–50 microns. General analysis of their properties and applications can be found in recent review [22]. The main physical parameter that influences the magnetic structure is magnetostriction $\left(\lambda_{s}\right)$, which can be controlled through composition and annealing. In CoFe based alloys, the magnetostriction is high and positive on the Fe-rich side ($\lambda_{s}\sim 10^{-5}$). Its value becomes small and negative when the Fe content is approximately 4–5 at%. In FeNi-alloy microwires, magnetostriction decreases with increasing Ni content. However, a higher Ni content also leads to a rapid decrease in both the saturation magnetization and Curie temperature [23,24]; consequently, nearly zero values of $\lambda_{s}$ correspond to transition to a paramagnetic state.

**Figure 4:**
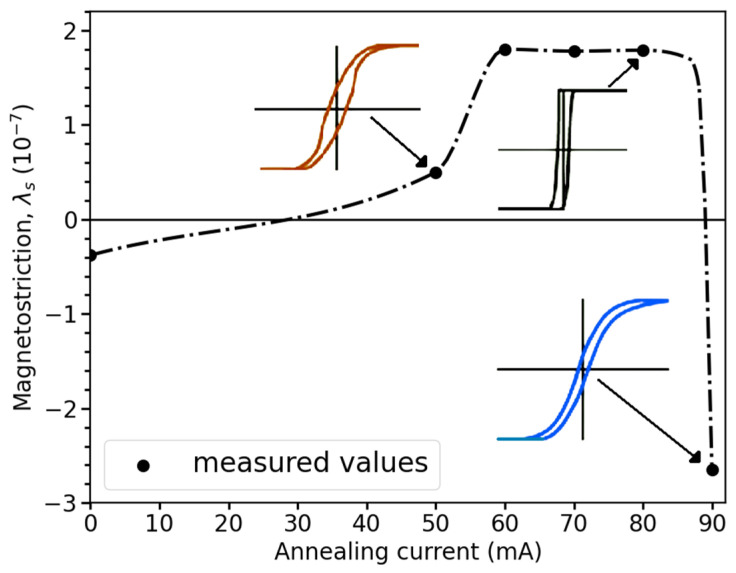
Variation in the magnetostriction of amorphous CoFeSiBCr microwires with the intensity of the annealing current, adapted from [50]. The change in the hysteresis loops for corresponding annealing conditions is shown. The dotted line is given for eye guiding.

The magnetostriction can be controlled by annealing. In CeFeSiBCr alloy microwires, current annealing makes it possible to tune magnetostriction from small negative values (~${-10}^{-7}$) to positive values, and then back to negative values as the annealing current is progressively increased. The resulting changes in magnetostriction, along with the corresponding modifications of the magnetic structure, are illustrated in Figure 4 [50].

Another important parameter affecting magnetic softness is the value and distribution of internal stresses within the metallic nucleus. These stresses originate from the rapid solidification of the metallic alloy itself, as well as from the difference in thermal expansion coefficients between the metallic alloy and the glass coating [51,52,53].

It is useful to consider the total magnetic energy $E_{m}$, which is contributed by averaged magnetocrystalline anisotropy $E_{cr}$, induced anisotropy $E_{u}$, and magnetoelastic anisotropy $E_{me}$ (inverse magnetostriction effect):

(6) $$E_{m}=E_{cr}+E_{u}{+E}_{me},$$

(7) $$E_{cr}=-K{\left(n_{k}\cdot m\right)}^{2}{,E_{u}=-K_{u}{\left(n_{u}\cdot m\right)}^{2},\;\;E}_{me}=-\frac{3}{2}\lambda_{s}\left(\hat{\sigma }m\right)\cdot m.$$

Here, $K,\;n_{k}$ and $K_{u},\;n_{u}$ are the magnitudes and directions of the crystalline and annealing-induced anisotropies, respectively, $m=M/M_{s}$ is the unit magnetization vector, and $\hat{\sigma }={\hat{\sigma }}_{in}+{\hat{\sigma }}_{ex}$ is the total stress tensor including internal stress ${\hat{\sigma }}_{in}$ occurring during fabrication and applied stress ${\hat{\sigma }}_{ex}$. In amorphous cobalt- based alloys the contribution of $E_{cr}$ could be larger than that of a magnetostrictive origin owing to clustering of Co-nanocrystals with high anisotropy. The parameter $\lambda_{s}$ also depends on stress; in particular, its dependence on axial stress $\sigma_{a}$ has the following form [23,54,55]:

(8) $$\lambda_{s}\left(\sigma_{a}\right)=\lambda_{s0}-\beta \sigma_{a}.$$

In Equation (8), $\lambda_{s0}$ is the saturation magnetostriction without any stress ($\hat{\sigma }=0)$ and the parameter β>0 is in the range of $(1-6)\cdot 10^{-10}$/MPa. The stress dependence of magnetostriction becomes important if $\lambda_{s0}$ is of the order of $10^{-7}$, which is typical for Co-based amorphous alloys. Then, as $\sigma_{a}$ increases, the negative contribution to $\lambda_{s}$ also increases, which could result in a sharp change in the direction of the easy anisotropy. The ability to modify magnetic structure by changing the direction of the easy axis through stress application is useful for creating highly sensitive mechanical stress sensing elements.

**Figure 5:**
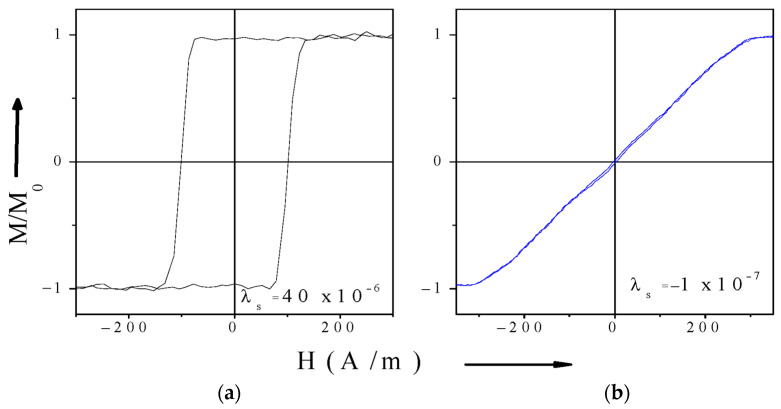
Hysteresis loops of magnetic microwires: (**a**) Fe_75_B_9_Si_12_C_4_ with positive magnetostriction (**b**) Co_67.1_Fe_3.8_Ni_1.4_Si_14.5_B_11.5_Mo_1.7_ with small negative magnetostriction [56].

The common viewpoint on the internal stress distribution in glass-coated microwires, confirmed by a number of indirect experiments such as measuring magnetic parameters during the gradual chemical etching of the glass coating or for microwires with different glass-coating thicknesses, is that the largest internal stresses present an axial orientation within almost the whole metallic nucleus volume [51,52,53]. Consequently, microwires with even the same composition and hence the same $\lambda_{s}$ values can present different magnetic properties. In general, however, due to the combination of internal tensile stress and magnetostriction with positive (Fe-rich) and negative (Co-rich) signs, amorphous FeCo-based alloy microwires coated in glass can exhibit both types of magnetizations discussed in Section 2, as shown in Figure 5 [56]. Therefore, they can be used in various sensors based on inductive spectroscopy.

Nanocrystalline glass-coated microwires could also be developed. The magnetic response is determined by the coexisting phases (i.e., the amorphous matrix with crystalline grains), which leads to small values of $E_{cr}$ and $\lambda_{s}$. For the FINEMET alloy, achieving vanishing overall magnetostriction maximizes the permeability and minimizes coercivity [57]. Interest also exists in developing nanocrystalline microwires with significant positive magnetostriction that exhibit magnetic bistability [58,59]. These can be achieved, for example, using nanocrystalline microwires based on FeNiMoB and FeCoMoB alloys. They exhibit magnetic bistability even in a nanocrystalline state because of the high positive magnetostriction in the amorphous and nanocrystalline states.

When microwires are used as sensing elements, the ferromagnetic alloy should have a high temperature of the transition to a paramagnetic state known as the Curie temperature, $T_{c}$. For temperature stability, the required value of $T_{c}\;$ is typically above 250 °C. To achieve this, the metalloids (Si and B) content must be less than 25% [24]. However, if microwires are used as temperature-sensing elements within the industrial temperature range (below 80 °C) or for biomedical applications, much lower $T_{c}$ values are required. This objective can be achieved by increasing the metalloid content; however, it should be noted that this results in a sharp decrease in saturation magnetization [24,60]. Special compositions with low $T_{c}$ and good magnetic properties at room temperature have been proposed [61,62]. For instance, adding Cr to CoFeSiB amorphous alloys decreases the Curie temperature due to antiferromagnetic pairing of Cr with Fe and Co atoms [63]. Addition of Cr in amount of 1 at% results in decrease in the Curie temperature by 24–25 °C. A sharper drop in $T_{c}$ was observed when the Cr amount in the alloy exceeded 10%. Additional decrease in the Curie temperature can be achieved by annealing which modifies the short-range ordering and can influence the atomic pairing [64,65]. For example, the Curie temperature of CoFeCr- allows can be further reduced by up to 7 °C by annealing at 250–300 °C.

### 3.1. Positive Magnetostrictive Microwires

Due to the predominantly axial stresses introduced during production, the domain structure of microwires with positive magnetostriction consists of axially magnetized domains surrounded by a thin layer of transverse closure domains. In glass-coated microwires, the axially magnetized domains may occupy almost entire volume since the axial stress from drawing is strong. As a result, the hysteresis loop is nearly rectangular. In some cases, a single axially magnetized domain is present, with reverse domains forming at the wire ends to reduce stray fields [22,56]. The magnetization effectively exhibits two stable states, ${+M}_{s}$ and ${-M}_{s}$ and switching between them occurs through the abrupt propagation of a reversal domain. This phenomenon is known as magnetic bistability, where the transition between these states takes place at a characteristic switching field, $H_{sw}$, accompanied by a large Barkhausen jump. In this case, $H_{sw}$ almost coincides with the coercive field, $H_{c}$.

Magnetic bistability has also been observed in nanocrystalline microwires. For FeNi MoB and FeCoMoB alloys, annealing the amorphous precursor at temperatures between 430 and 600 °C results in the formation of a stable nanocrystalline structure composed of α-FeCo crystalline grains embedded in a residual amorphous matrix. The magnetostriction of the FeCoMoB nanocrystalline microwire was found to be anisotropic [66], and this anisotropy promotes the formation of a single-domain structure, thereby leading to magnetic bistability.

The switching process has been found to depend on the wire geometry as well as on external factors such as mechanical stress and temperature, which can be exploited for sensing applications. The switching field is determined by the nucleation of the reversal domain and its depinning from defects or potential wells formed by stress inhomogeneities. Therefore, $H_{sw}$ is proportional to the surface energy ϵ of the domain boundary and its spatial distribution. It is reasonable to suggest that

(9) $$H_{sw}\propto \frac{\epsilon }{{\mu_{0}M}_{s}},\;\epsilon =\pi \sqrt{AK}\;,$$

where K is the total anisotropy energy density defined by Equation (7) within the wall and *A* is the exchange stiffness. For microwires with larger magnetostriction, the main contribution to the anisotropy energy originates from magnetoelastic interactions. In this case $K=(3/2{)\lambda }_{s}\sigma$, where σ is the axial stress. Several studies have confirmed that $H_{sw}$ closely follows the $\sigma^{1/2}$–law [18,66,67].

The switching field can also exhibit temperature sensitivity. This behavior has been observed within practical temperature ranges in nanocrystalline microwires, as well as in microwires with a low Curie temperature. In alloys with early nanocrystalline state (with a low nanocrystal volume), a sharp increase in $H_{sw}$ is observed near the Curie temperature of the amorphous phase. The nanocrystalline grains lose their exchange interaction with each other and, due to this decoupling, behave as single-domain particles [68,69]. This behavior can occur within a narrow temperature range, 20–45 °C in FeCoMoBCu wires with a high molybdenum content after appropriate annealing, as a result of the very low Curie temperature of the residual amorphous matrix. Moreover, the uncoupled nanocrystalline grains, owing to their weak mutual interactions, act as pinning centers for domain wall propagation. As a consequence, the switching field increases sharply as the applied temperature approaches the Curie temperature of the residual amorphous matrix. In nanocrystalline Fe_71.16_Mo_9.85_B_18_Cu_0.99_ microwires magnetic bistability was achieved after specially performed annealing under stress of 309 MPa above the crystallization temperature ($T_{cr}$∼461 °C) at 500 °C for 1 h and subsequent slow cooling. For low frequencies of the magnetizing field of 400 Hz, the values of $H_{sw}$ increases almost twice when temperature increases from 20 to 45 °C, demonstrating an almost linear dependence [69].

In amorphous microwires made of low Curie temperature alloys, a decrease in $H_{sw}$ can be expected in the proximity to $T_{c}$. In this case in Equation (9), $A\propto M_{s}^{2},\;\lambda_{s}\propto M_{s}^{n}$, n ranges from 2 to 3 according to the classical anisotropy model of localized spins [70]. Therefore, $H_{sw}\;\propto$ $M_{s}^{n/2}$ and it will decrease with temperature when approaching to $T_{c}$.

**Figure 6:**
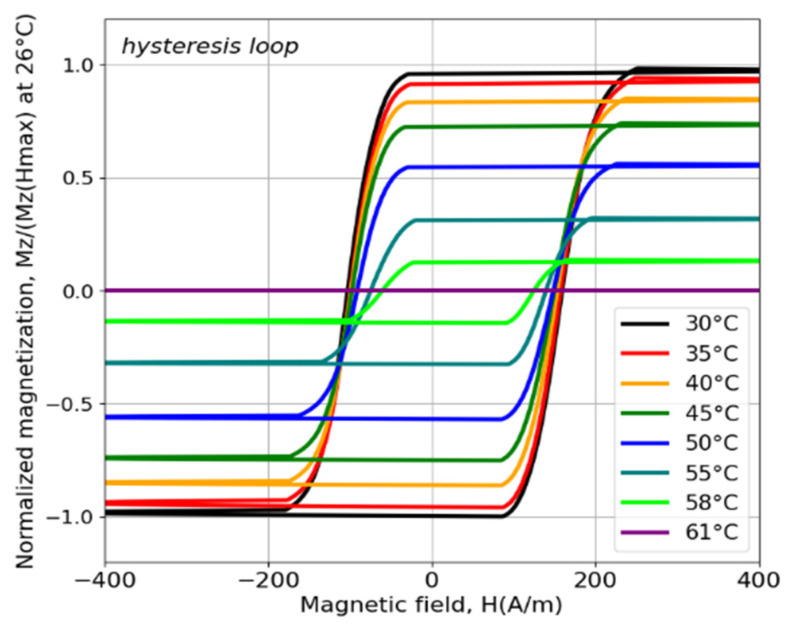
Hysteresis loops at different temperatures for microwires in a glass coating comprising the following composition: Co_64.82_Fe_3.9_B_10.2_Si_12_Cr_9_Mo_0.08_. Adapted from [72].

Amorphous microwires with a high content of Cr between 8 and 10 at% have a Curie temperature in the range 60–100 °C [63,71]. For example, microwires with the composition Co_64.82_Fe_3.9_B_10.2_Si_12_Cr_9_Mo_0.08_, exhibit $T_{C}$~61 °C. After annealing, the Curie temperature can decrease to 57 °C [72]. This decrease results from changes in the compositional short-range ordering, leading to stronger antiferromagnetic coupling between Fe-Cr and Co-Cr atoms. Despite the high content of Co, the saturation magnetostriction is positive but relatively small 2.8 × 10^−7^. The hysteresis loops shown in Figure 6 exhibit a nearly rectangular shape with $M_{r}/M_{s}\sim 1$, even at temperatures close to $T_{c}$, indicating that the easy anisotropy remains axial. However, these microwires do not display true bistable behavior.

The coercivity mechanisms can be more complex depending on gradient of internal stress distribution and defects, leading to deviations from Equation (9), particularly when the wire has almost a rectangular loop but lacks bistable behavior [73]. For example, coercivity field in microwires with addition of tungsten used to decrease its Curie temperature demonstrated a complex temperature dependence [74]. In the case of temperature sensing, harmonics detection is therefore more suitable. The area under the pulse $S=\int V\left(t\right)dt$ is proportional to $M_{s}$. Consequently, the harmonics amplitudes show pronounced alterations near to $T_{c}$, as illustrated in Figure 1e, which presents the harmonics amplitudes for two values of S.

### 3.2. Negative Magnetostriction Microwires

**Figure 7:**
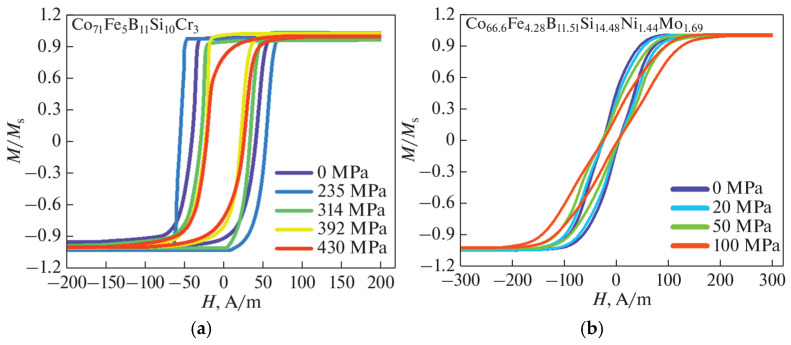
Tensile stress effect on the magnetization loops in amorphous wires of the composition (**a**) Co_71_Fe_5_B_11_Si_10_Cr_3,_ *D*/*d* = 29/25 and (**b**) Co_66.6_Fe_4.28_B_11.51_Si_14.48_Ni_1.44_Mo_1.69_, D/d = 35/25 µm. In the case of (**b**) the wire has a thicker glass coating and stronger magnetostrictive contribution to anisotropy. Adapted from [37].

Magnetic microwires with low negative magnetostriction typically have a circular anisotropy and a so-called bamboo domain structure at least in the outer region. This is related to the coupling of a negative magnetostriction $\lambda_{s}$ with internal tensile stresses. If this stress is predominant, the inner region with axial magnetization is small as evident from hysteresis loops with small remanence magnetization and coercivity field, shown in Figure 5b. However, in some cases, due to the existence of Co nanocrystal clustering, which has high magnetic anisotropy, the overall anisotropy becomes axial ($K=\left(4.2-5.5\right)\cdot 10^{5}\;J/m^{3}$ for hcp Co). This was demonstrated for a number of compositions, such as Co_71_Fe_5_B_11_Si_10_Cr_3_. In this case, the hysteresis loop of the as-prepared microwire is almost rectangular. The application of external stress increases the magnitude of magnetostriction, making it more negative according to Equation (8). The contribution of the magnetoelastic anisotropy increases and alters the direction of the anisotropy easy axis: it aligns closer to the circumference due to coupling between negative magnetostriction and axial stress $\sigma_{a}$, as shown in Figure 7a. For wires of slightly different composition with larger magnetostriction or internal stress due to impact of glass coating, a circumferential anisotropy is observed without the applied stress, as shown in Figure 7b. From the inclined hysteresis loop, it is possible to estimate the effective circular anisotropy field $H_{K}$, whose value increases monotonically from 70 A/m to 200 A/m at $\sigma_{ex}$ = 100 MPa.

Amorphous microwires with negative magnetostriction can be produced from alloys with a low Curie temperature. For example, a microwire of Co_27.4_Fe_5_B_12.26_Si_12.26_Ni_43.08_ composition, which has a Curie temperature of 48 °C and magnetostriction of $-4.2\cdot 10^{-7}$ [65]. These microwires exhibit an inclined hysteresis loop, which is indicative of circular anisotropy due to negative magnetostriction. The shape of the hysteresis loops remains unchanged when approaching $T_{c}$, which suggests that the magnetostriction remains negative. However, the slope of inclination decreases with increasing temperature due to a decrease in susceptibility.

In negative magnetostrictive wires with a circular domain structure the passing current results in the movement of circular domains. A circular hysteresis loop ($M_{\varphi }-H_{\varphi }$) can be obtained by integrating the voltage induced by the change in the circumferential magnetic flux. It has been demonstrated in a number of works that such a loop can exhibit a nearly rectangular form, and that movement of a single circular wall can be observed [75,76,77]. This indicates that the process of circular magnetization may be facilitated by large Barkhausen jumps of circular domain walls, resulting in a bistable type of circular magnetization. The application of a dc magnetic field will have a smoothing effect on circular magnetization by stimulating the rotational processes.

## 4. Glass-Coated Microwires for Nonlinear Inductive Sensing Elements

### 4.1. Sensing Elements Using Microwires with Positive Magnetostriction

In the case of bi-stable wires, where the induced pulse position is clearly identified as the switching field or switching time, various wireless sensors have been developed on this principle. To assure high sensitivity to the mechanical stress, microwires of alloy composition with high glass-forming ability, high saturation magnetization and low switching field should be selected. High sensitivity to temperature is achieved in nanocrystalline alloys and alloys with low Curie temperature. In all cases, to support bistability of magnetic properties, positive magnetostriction with moderate values is required.

In the earliest attempts to demonstrate the method, the bistable Fe_38.5_Ni_39_Si_7.5_B_15_ microwires were used for stress monitoring in composite fiberglass layered materials [17]. The microwire sample of different length (1–4 cm) was positions between the layers. In all cases, the stress response of the wires was observed and compared with various applied stresses, which changed differently over time. A reasonable agreement was observed between continuously applied stress and measured time response when the switching events occurred. However, depending on position and length, different responses were observed and in some cases with considerable deviation from the applied forces. This was explained by structural differences and lack of wire-material bonding.

Bistable microwires have been found to be potentially suitable for measuring mechanical strains in different types of structures such as concrete structures and various composite materials. The size of the microwire is smaller than the size of structural elements, such as sand grains that form the concrete or reinforcing fibers. Hence, the microwire does not play the role of the defect in materials.

It was verified the feasibility of stress monitoring for concrete materials using bistable microwires [78,79,80,81]. A sensing element consisted of a magnetic microwire embedded in a cement-based material to ensure good coupling of the microwire to the concrete-based material, where this sensor would be embedded. The compressive stress dependence of the measured switching field showed the decrease due to the compensation of tensile stress applied on metallic nucleus by glass-coating and followed the square root law according to Equation (9). However, in many other switching field measurements, a nearly linear dependence on stress was observed, which was attributed to a more complicated domain structure.

Relatively thick Fe_71.7_B_13.4_Si_11_Nb_3_Ni_0.9_ glass-coated amorphous microwires (d = 103 μm and D = 158 μm) were employed to fabricate sensor arrays embedded into a cement mortar for stress/strain measurements [79]. The cement-based composite’s mix proportions were selected to provide a compressive strength comparable to that of the aggregates utilized. This configuration was proposed for measuring the weight of individual vehicles while in motion. During cyclic loading, a hydraulic press was employed to apply the stress. The excitation coil was positioned externally, and the pickup winding was embedded around the mortar cylinder containing the microwires to perform the measurements. The test results showed good agreement between conventional gauge sensors and the microwire-based sensors.

**Figure 8:**
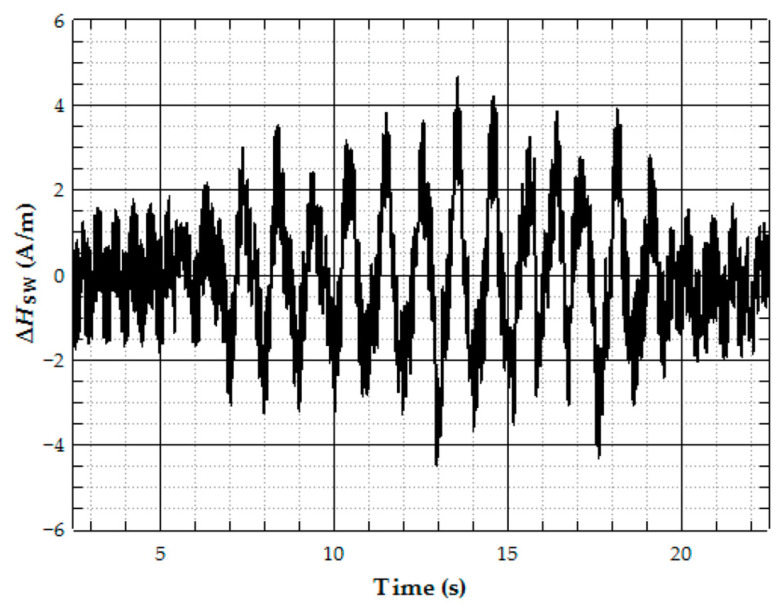
Record of the Fe_77.5_Si_7.5_B_15_ microwire switching field deviation during the aircraft swing movement [80].

An Fe_77.5_Si_7.5_B_15_ microwire was employed as a tensile-stress sensor to evaluate stresses arising in aircraft wings during operation [80]. The experiments were carried out on ultralight aircraft provided by Incoff Aerospace. Prior to field testing, the sensor sensitivity was determined under laboratory conditions to be approximately 0.2 A·m^−1^·MPa^−1^ for short wire samples with a length of 2 cm. These samples were mounted on the upper surface of the aircraft wing and covered with a glass-fiber fabric. Figure 8 presents a representative measurement. In this experiment, the aircraft was manually oscillated about its longitudinal axis, producing the maximum tensile stress in the main wing spar.

Non-contact methods of measurements of stress/strain and temperature with the use of bistable microwires were proposed for medical applications [30,31,32,69,82]. Due to their Pyrex glass coating, microwires are biocompatible, as confirmed by several studies. In [83], cytotoxicity tests were conducted using the human hepatocellular carcinoma cell line (Huh7). Fe- and Co-based microwires with a metal core diameter of 20–30 µm and a glass coating thickness of 3–5 µm were placed in a nutrient medium alongside the cell culture. After a 24 h incubation, propidium iodide staining was used to identify dead cells, and the labeled cells were analyzed using epifluorescence microscopy. In [84], the glass-coated microwires were evaluated with a human embryonic fibroblast cell culture. Cell viability was assessed using fluorescent staining with ethidium bromide and the MTT assay. No significant differences were observed between cultures with and without microwires, and these tests were conducted in the presence of a magnetic field. These results confirm that the microwires are non-toxic to cell cultures.

An Fe_76_Si_9_B_10_P_5_ (d = 28 µm, D = 53 µm) amorphous microwire was used for intracranial pressure measurements, which are crucial for detecting secondary brain injury [82]. In the experiments, a skull model with a water-filled balloon representing the cerebral membrane was used. The elastic properties of the balloon closely matched those of the cerebral membrane, allowing the microwire fixed to the balloon to serve as a sensing element for intracranial pressure. The induction and detection coils were positioned outside the skull, close to the microwire. The switching time of the microwire increased monotonically with the applied pressure, exhibiting a sensitivity of 24 µs/kPa and a full-scale range up to 2700 Pa, corresponding to slightly elevated intracranial pressure.

The feasibility of measuring stress applied to bones was also demonstrated using bistable microwires of various compositions, for example, Fe_42.6_Ni_34.9_Si_7.5_B_15_. Measurements were taken under both compression and stress-release conditions [30].

The use of bistable microwires for stress and pressure measurements in organs can be viewed in the context of recent progress in ultrasonic sensing. Wearable ultrasonic devices can be employed not only for imaging, but also for monitoring physiological parameters such as blood pressure and shear-wave velocities during muscle motion, thereby providing dynamic information on muscle mechanical properties [85]. However, the development of such devices still faces several challenges, including the need to improve piezoelectric materials, whose performance degrades as the size of the vibrating element decreases.

Microwires with temperature-dependent switching fields or with a low Curie temperature were proposed for monitoring inflammatory processes after implantation. Nanocrystalline microwires of the composition Fe_77_Cr_1_Mo_6_B_15_Cu_1_ which exhibit a strong increase in $H_{SW}$ within a narrow temperature range of 20–45 °C, were tested for non-contact temperature measurements in the cranium near the TiAl_6_V_4_ implant [69]. The measurements were taken using a skull model with a titanium implant. To sense the temperature inside the cranium, the microwire was attached to the internal surface of the Ti implant using an adhesive tape, while the switching field was detected externally using a standard inductive setup. Water at approximately 45 °C was introduced into the model of the cranium. As the temperature was cooling, it was monitored using a thermometer. The time variation in induced voltage maxima (caused by Burkhausen jump in microwires) between the minimum and maximum temperatures was 65.77 μs, corresponding to a measurement sensitivity of 10.8 μs/°C. This experiment shows that it is possible to sense the temperature in vivo even through the Ti implant.

**Figure 9:**
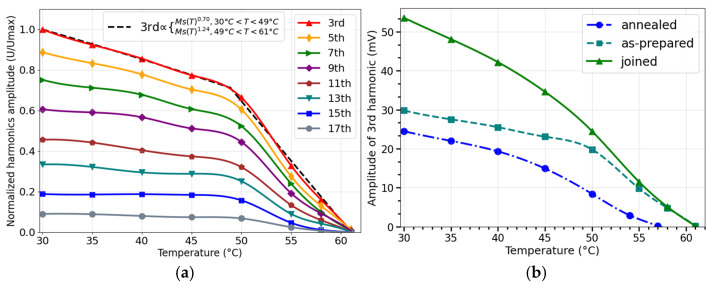
In (**a**), Temperature behavior of harmonics amplitudes for as-prepared microwire of the composition Co_64.82_Fe_3.9_B_10.2_Si_12_Cr_9_Mo_0.08_ (total diameter D/d=29.2/18μm). In (**b**), the temperature dependence of the 3-rd. harmonic amplitude for two wire samples- as-prepared and annealed at 50 mA, joined together. Adapted from [72].

The switching field measurement can be challenging since the positioning of the excitation/detection coils requires precision. A full spectral analysis can overcome this issue because the average ratio of several harmonics is obtained independently of coil arrangement and detection distance. The spectral response of microwires with a low Curie temperature offers potential benefits for temperature sensing in biomedical applications. The induced voltage pulse contains odd harmonics of the excitation frequency $f_{0}$, and their amplitudes decrease with increasing temperature, approaching $T_{c}$. For lower harmonics numbers, this decrease is almost proportional to $M_{s}\left(T\right)$. Using a microwire of composition Co_64.82_Fe_3.9_B_10.2_Si_12_Cr_9_Mo_0.08_ with $T_{c}\sim$61 °C a sharp decrease in harmonics amplitude was observed within a temperature range of 50–61 °C, as shown in Figure 9 [72]. Therefore, this method provides a temperature dependence similar to that obtained from $H_{c}\left(T\right)-$ measurements but allows the use of multiple measurement parameters such as higher order harmonics amplitudes and their combinations for temperature evaluation, along with specific noise reduction techniques based on lock-in detection. Additionally, using different wires with slightly varied Curie temperatures, achieved through annealing, provides more flexibility in extending the sensitive temperature range. As evident from Figure 9b, the temperature-sensitive spectral responses of two microwires (one as-cast and one annealed with a Curie temperature of 57 °C) positioned 1–2 mm apart have a temperature-sensitive range of 30–61 °C.

### 4.2. Sensing Elements Using Microwires with Negative Magnetostriction

**Figure 10:**
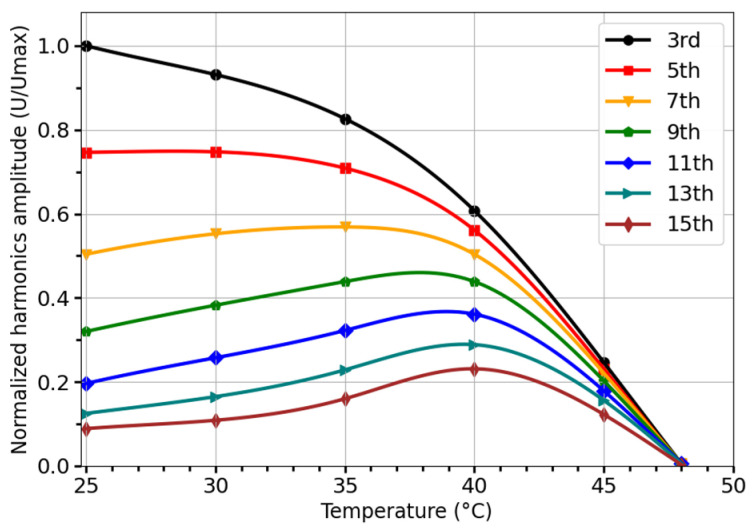
Temperature dependence of harmonics amplitudes for microwire of the composition Co_27.4_Fe_5_B_12.26_Si_12.26_Ni_43.08_ (a total diameter of D = 37 μm and a metal core diameter of d = 31 μm).

Amorphous microwires with negative magnetostriction and a low Curie temperature can also be used as wireless temperature sensors based on spectral analysis. For example, the harmonics amplitudes of a microwire with the composition Co_27.4_Fe_5_B_12.26_Si_12.26_Ni_43.08_ experience a sharp decrease as the temperature approaches  = 48 °C, as shown in Figure 10. The range in which this sharp decrease occurs varies with harmonic order, with higher order harmonics exhibiting a non-monotonic dependence on $T_{c}$. For lower order harmonics, the range is between 35 °C and 48 °C, while for higher harmonics, it is between 40 °C and 48 °C. For lower-order harmonics this decrease is almost proportional to $M_{s}\left(T\right)$. For example, the third harmonic decreases as $M_{s}^{1.1}$.

**Figure 11:**
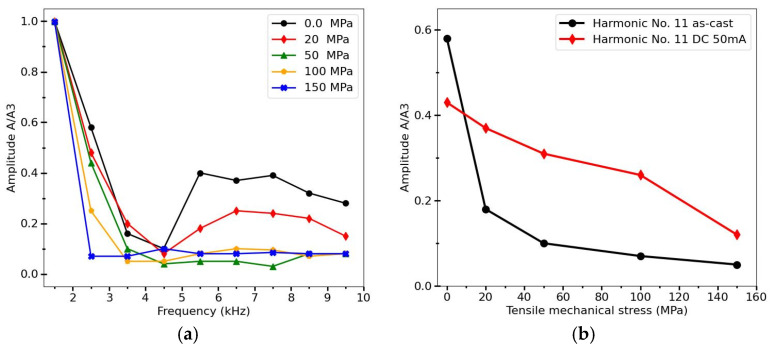
(**a**) Harmonics amplitudes of Co_66.6_Fe_4.28_B_11.51_Si_14.48_Ni_1.44_Mo_1.69_ amorphous microwire magnetized by flat coil which generates a magnetic field of 325 A/M in the center for different tensile stresses. (**b**) Stress dependence for a particular harmonic (No. 11) for as–cast and annealed microwire (50 mA, 10 min). Magnetizing field frequency was 500 Hz. Amplitudes are normalized to the value of the 3d harmonics (A/A3). Adapted from [37].

In the case of negative magnetostriction microwires typically having circumferential easy magnetization and exhibiting hysteresis loops of the type shown in Figure 1b and Figure 5b, it is possible to use spectral analysis to measure mechanical stress, since the application of stress alters the effective anisotropy and, consequently, the susceptibility, as seen from the hysteresis loops in Figure 7. This was proposed for stress measurements inside composite materials using planar magnetizing and detection coils [37]. In such experiments, a microwire with the composition Co_66.6_Fe_4.28_B_11.51_Si_14.48_Ni_1.44_Mo_1.69_ (D/d = 35/25 microns), with magnetostriction of approximately $-2\cdot 10^{-7}$, and with an inclined hysteresis loop shown in Figure 7b, was used. The microwire was magnetized by a flat coil built on a PCB, which produced a magnetizing field up to 875 A/m, sufficient to magnetize the wire. The differential detection coils were also located on the same coil [86]. The induced voltage pulse becomes broader under application of tensile stress which is reflected in the change in the harmonic spectrum, as demonstrated in Figure 11. Clearly, the amplitudes of the harmonics do not exhibit monotonic behavior as the harmonic order n increases, which is typical of an inclined magnetization curve approaching saturation (compare with Figure 1f). The amplitudes decrease sharply with increasing stress above 50 MPa as the susceptibility drops. With a further increase in mechanical stress, these changes become insignificant. To extend the stress-sensitivity range, the wire can be annealed to enhance circular anisotropy, as shown in Figure 11b.

**Figure 12:**
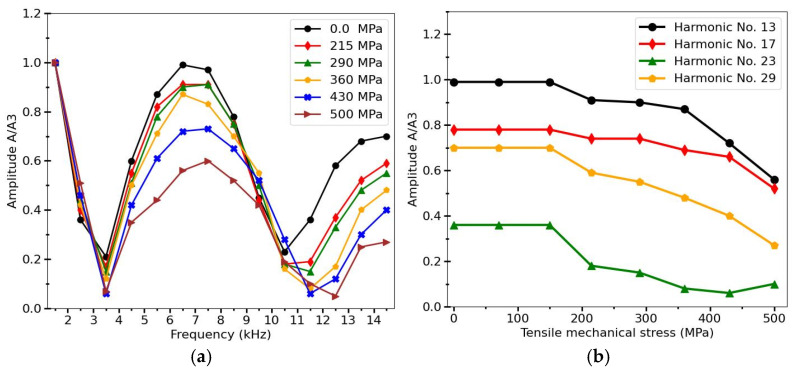
(**a**) Harmonics amplitudes of Co_71_Fe_5_B_11_Si_10_Cr_3_ amorphous microwire magnetized by flat coil which generates a magnetic field of 325 A/M in the center for different tensile stresses. (**b**) Stress dependences for particular harmonics (No. 13, 17, 23, 29). Magnetizing field frequency was 500 Hz. Amplitudes are normalized to the value of the 3d harmonics (A/A3). Adapted from [37].

It is interesting to consider the stress influence on the harmonic spectrum in microwires having a negative magnetostriction but a nearly rectangular hysteresis loop in as-prepared state. An example of such magnetic hysteresis is shown in Figure 7a [37,87]) for Co_71_Fe_5_B_11_Si_10_Cr_3_ microwire. In this case, the stress-sensitive range is quite different: no changes in harmonic amplitudes are observed at stresses below 150 MPa, as seen in Figure 12. This correlates with the shape of the hysteresis loops, which remains rectangular until higher stress is applied. For higher stresses, the amplitude variations exceeding 100% can be achieved for certain harmonics, as for harmonics with n = 29 (as shown in Figure 12b). It should be noted that dependencies of harmonic amplitudes on mechanical stresses are observed even for normalized amplitudes. In particular, this was observed when using the 3rd-harmonics amplitude for normalization, as shown in Figure 11 and Figure 12. For practical applications, this allows the influence of various design factors and the distance of measurements to be avoided.

**Figure 13:**
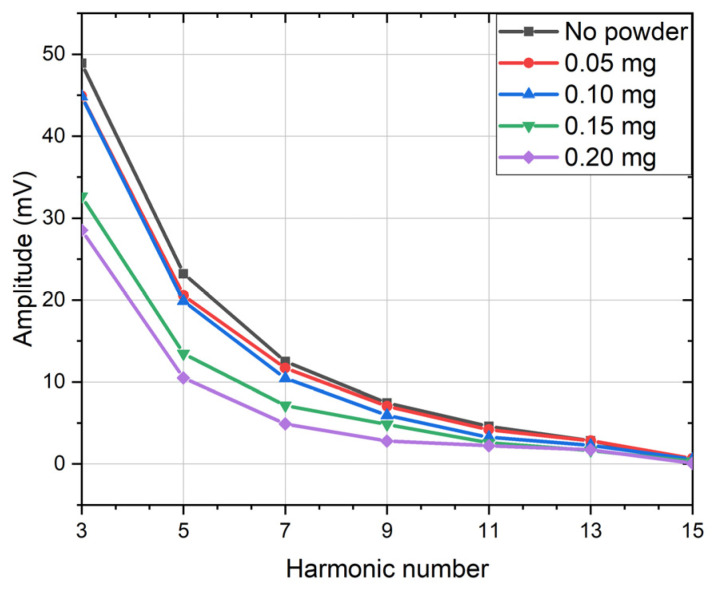
Harmonic response of a Co_66.6_Fe_4.28_B_11.51_Si_14.4_Ni_1.44_Mo_1.69_ amorphous microwires annealed by a current of 15 mA in the presence of Ba-hexaferrite nanoparticles of different mass, when a current of 15 mA and a frequency of 100 kHz is passing.

In Co-based microwires with an easy circumferential magnetization and a well-defined circular domain structure characterized by a hysteresis loop shown in Figure 5b, an ac current of sufficient magnitude has been shown to induce the movement of circular domain walls, generating a measurable voltage across the wire at frequencies in the 10 kHz range [75,77]. Given the irreversible nature of domain wall motion, the change in the circular magnetization $M_{\varphi }$ caused by the field $H_{\varphi }$, produced by the current, is strongly nonlinear. Consequently, the induced voltage signal comprises higher harmonics of the excitation source. The output signal correlates with the nonlinearities present in $M_{\varphi }$ vs. $H_{\varphi }$ dependence and varies in response to the external stimuli that influence this relationship such as magnetic fields or magnetic particles. The change in harmonic response in the presence of Ba-hexaferrite particles is demonstrated in Figure 13. The particles were prepared using the hydrothermal method, which requires post-processing to produce hexaferrite phase. These particles demonstrated weak magnetic properties immediately after hydrothermal treatment and therefore produced little effect on the spectrum. However, after annealing and the formation of the hexaferrite phase with high remanence magnetization, their presence caused a suppression of the harmonics’ amplitude proportional to their quantity.

## 5. Conclusions

Glass-coated microwires are ideal materials for constructing miniaturized sensors (e.g., temperature, stress and position sensors), including contactless sensors, which can be embedded in various structures and materials for engineering and biomedical applications. The operation of the sensors is based on nonlinear magnetization dynamics, whereby the induced voltage response correlates with the nonlinearities present in the magnetization versus magnetic field dependence, which varies in response to an external stimulus affecting this dependence. The preferable detection mechanism when using microwires exhibiting magnetic bistability is to detect the switching field or time at which large Barkhausen jumps occur. However, measuring the switching field can be challenging. Full spectral analysis of the induced voltage using microwires with a low Curie temperature has potential benefits for temperature sensing, particularly in biomedical applications.

Various frequency-mixing techniques have been investigated as a means of improving signal extraction and overall sensor performance. This technique is mainly applied to magnetic particle detection, but it has a potential to be used for property measurements.

For microwires with circular anisotropy and inclined hysteresis loops, the harmonic spectrum depends heavily on the circular anisotropy characteristic, which can be used for mechanical stress and temperature measurements. The readout schemes are based on higher-order harmonic analysis, which is implemented via lock-in amplification and can provide enhanced sensitivity and distance independent detection.

## Data Availability

The data that support the authors’ findings, mainly used for Figure 6, Figure 7, Figure 9, Figure 10, Figure 11, Figure 12 and Figure 13, are available on request from the corresponding author, L.V.P.

## Abbreviations

The following abbreviations are used in this manuscript:

MI	Magnetoimpedance
DC	Direct Current
*Ms*	Saturation Magnetization
*Hsw*	Switching field
*Hc*	Coercive field
MPI	Magnetic Particle Imaging
PCB	Printed Circuit Board
*Mφ*	circular magnetization
*Hφ*	circular field
*FFT*	Fast Fourie Transform
